# Expression of Osteoarthritis Marker YKL-39 is Stimulated by Transforming Growth Factor Beta (TGF-beta) and IL-4 in Differentiating Macrophages

**Published:** 2008-02-14

**Authors:** Alexei Gratchev, Christina Schmuttermaier, Srinivas Mamidi, LiMing Gooi, Sergij Goerdt, Julia Kzhyshkowska

**Affiliations:** Department of Dermatology, University Medical Centre Mannheim, Ruprecht-Karls University of Heidelberg, Mannheim, Germany D-68167

**Keywords:** osteoarthritis, chitinase, YKL-39, macrophage, TGF-beta, IL-4

## Abstract

YKL-39 is a Glyco_18 domain containing chitinase-like protein which is currently recognized as a biomarker for the activation of chondrocytes and the progress of the osteoarthritis in human. YKL-39 was identified as an abundantly secreted protein in primary culture of human articular chondrocytes. Two biological activities of YKL-39 might contribute to the disease progression. One is the induction of autoimmune response and second is the participation in tissue remodeling. Other mammalian chitinase-like proteins including chitotriosidase, SI-CLP, YKL-40 and YM1 are expressed by macrophages in various pathological conditions. In contrast, YKL-39 was never reported to be produced by macrophages. We used in vitro model of human monocyte-derived macrophage differentiation to analyse regulation of YKL-39 expression. Expression of YKL-39 was examined by real-time RT-PCR. CD14+ MACS sorted human monocytes differentiated for 6 days under different stimulations including IFNγ, IL-4, dexamethasone and TGF-β. We found that both IL-4 and TGF-β have weak stimulatory effect on YKL-39 expression in all donors tested (3.2 ± 1.7 fold, p = 0.006 and 6.3 ± 3.1 fold, p = 0.014 respectively). However the combination of IL-4 and TGF-β had strong stimulatory effect on the expression of YKL-39 in all analysed individual macrophage cultures (34 ± 36 fold, p = 0.05). IFN-γ did not show statistically significant effect of YKL-39 mRNA expression. Presence of dexamethasone almost completely abolished the stimulatory effects of IL-4 and TGF-β. In summary, we show here for the first time, that human cells of monocyte origin are able to produce YKL-39. Maturation of monocyte derived macrophages in the presence of Th2 cytokine IL-4 and TGF-β leads to the strong activation of YKL-39 expression. Thus elevated levels of YKL-39 observed during chronic inflammations can not be attributed solely to the activity of chondrocytes. In perspective, YKL-39 might serve as a useful biomarker to detect macrophage-specific response in pathologies like tumour, atherosclerosis and Alzheimer disease.

## Introduction

YKL-39 (homo sapiens chitinase-like 2, CHI3L2) is currently recognized as a biochemical marker for the activation of chondrocytes and the progress of the osteoarthritis in human (Knorr et al. 2003). YKL-39 is secreted human chitinase-like protein which contains Glyco_18 domain with no catalytic activity but with putative lectin properties (Funkhouser et al. 2007; Kzhyshkowska et al. 2007). YKL-39 was originally identified as an abundantly secreted protein in primary culture of human articular chondrocytes (Hu et al. 1996). In contrast to its closest homologue YKL-40, YKL-39 is not a glycoprotein. Although the number of aminoacids was slightly bigger in YKL-39, apparent higher MW of YKL-40 is due to the presence of carbohydrate (Hu et al. 1996). YKL-39 accounted for 4% and YKL-40 for 33% of the secreted protein in chondrocyte-conditioned medium. Despite the high homology on the protein level (more than 50%), the radioimmunoassay developed for the detection of YKL-40 in serum and in tissues, was shown to detect exclusively YKL-40 but not YKL-39 (Hu et al. 1996).

Comparison of the expression of YKL-39 and YKL-40 in osteoarthritic cartilage revealed, that YKL-39 mRNA is significantly up-regulated in cartilage of patients with osteoarthritis (OA) versus normal subjects, while no significant up-regulation was detected for YKL-40 mRNA in OA cartilage (Steck et al. 2002). Study of Knorr et al. showed that normal human chondrocytes express mRNA for both YKL-39 and YKL-40. However while the expression of YKL-39 was upregulated both in early degenerative and late stage osteoarthritis, the expression of YKL-40 was downregulated during the progression of osteoarthritis (Knorr et al. 2003). Proteomic analysis identified YKL-39, but not YKL-40 to be secreted by human osteoarthritic cartilage in culture (De Ceuninck et al. 2005).

The closest homologue of YKL-39, YKL-40 has multiple biological activities, including a growth-promoting effect on human synovial cells, skin and fetal lung fibroblasts as well as immuno-modulatory effects on monocytes and T-cells, while examination of the biological effects of purified YKL-39 remains to be done (reviewed in Kzhyshkowska et al. 2007). However two physiological activities of YKL-39 might contribute to the disease progression. First one is the induction of autoimmune response (Du et al. 2005; Sekine et al. 2001; Tsuruha et al. 2002), and second is predicated participation in tissue remodeling.

Several mammalian Glyco_18 domain containing proteins are expressed by macrophages in various physiological situations (Kzhyshkowska et al. 2006; Kzhyshkowska et al. 2007). Thus, enzymatically active chitotriosidase is expressed by mature monocyte derived macrophages, lung macrophages and Gaucher cells (Boot et al. 1999; Di Rosa et al. 2005; Malaguarnera et al. 2005; Renkema et al. 1995; van Eijk et al. 2005). Gaucher cells are abnormal lipid-laden macrophages formed in tissues of Gaucher Disease patients. Gaucher cells can be classified as a variation of alternatively activated macrophages (Boven et al. 2004). Thus chitotriosidase is used as a highly specific serum biomarker for lysosomal storage disorder (Aerts et al. 2005). Macrophages seem to be the source of secreted YKL-40, which serum levels are elevated in numerous tumours, chronic and acute inflammations and during fibrosis progression. Catalytically inactive SI-CLP (stabilin-1 interacting chitinase-like proteins) and murine YM1/2 are expressed by macrophages in Th2-environment (Hogenesch et al. 2006; Kzhyshkowska et al. 2006; Raes et al. 2002; Webb et al. 2001).

In contrast to other mammalian Glyco_18 domain containing proteins, YKL-39 production is clearly documented only for chondrocytes and synoviocytes (Hu et al. 1996). Recently we performed comparative quantitative analysis using real-time RT-PCR and demonstrated that YKL-40 was strongly upregulated by IFNγ, while YKL-39 mRNA was expressed on a very low level in macrophages differentiated in the presence of IFNγ, IL-4 or dexamethasone (Kzhyshkowska et al. 2006). To our knowledge there is only one report suggesting that YKL-39 can be expressed by cells of monocyte origin. Messenger RNAs for both YKL-39 and YKL-40 were strongly upregulated in the brain of patients with Alzheimer disease, and this fact was attributed to the alternative activation of microglial macrophages during the course of the disease (Colton et al. 2006). However specific stimuli responsible for the induction of YKL-39 were never demonstrated.

Here we present the evidence, that macrophages are able to produce YKL-39 in response to TGF-β stimulation. Analysis of human primary monocyte-derived macrophages revealed, that their differentiation in the presence of TGF-β and Th2 cytokine IL-4 leads to strong statistically significant activation of YKL-39 mRNA. Single stimulations with IL-4 alone and TGF-β alone had only slight stimulatory effect, while dexamethasone inhibited expression of YKL-39 mRNA. These data suggest, that elevated levels of YKL-39 observed during chronic inflammation can be assigned not only to the activation of synovial cells, and macrophages can contribute to the YKL-39 production if IL-4 and TGF-β are present concomitantly. Elevated level of YKL-39 can be also expected in pathologies like tumour, neurodegenerative diseases and atherosclerosis.

## Materials and Methods

### Monocyte isolation and cultivation of macrophages

The isolation and cultivation of human monocytes/macrophages was done as described (Gratchev et al. 2004; Gratchev et al. 2005; Gratchev et al. 2006). The cells were purified from individual buffy coats. Buffy coats were diluted with Ca^2+^- and Mg^2+^- free phosphate-buffered saline (PBS) (Biochrom) at a ratio 1:1. A total of 35 ml of diluted buffy coats were layered on top of 15 ml Ficoll-Paque (Biochrom, Berlin, Germany) in a 50-ml Leucosep tube (Greiner, Fickenhausen, Germany). After 30 min of centrifugation in a swing out rotor (Beckman Coulter, Fullerton, CA, U.S.A.) at 650 × g, peripheral blood mononuclear cells (PBMC) were collected from the Ficoll serum interphase. PBMCs were washed two times with PBS (Biochrom). The Percoll gradient was preformed by centrifugation of freshly prepared Percoll (13.5 ml Percoll (Pharmacia, Freiburg, Germany), 1.5 ml 10 × Earle’s Minimal Essential Medium (MEM), 15 ml Spinner’s medium) at 12000 × g for 10 min at 20 °C without breaks in a F34-6-38 rotor (Eppendorf, Hamburg, Germany). Five to 8 × 10^8^ PBMCs were layered on top of the Percoll gradient and centrifuged at 650×g for 30 min at 20 °C without breaks. The upper layer, containing 60%–80% monocytes was collected and washed three times with PBS. The cell population obtained after Percoll gradient centrifugation was subjected to CD14+ magnetic cell sorting using monocyte isolation kit (Miltenyi Biotech, Bergisch Gladbach, Germany), resulting in 92–98% monocyte purity, controlled by flow cytometry. Macrophages were cultured at 1 × 10^6^ cell/mL in X-vivo 10 serum free medium (Cambrex, Verviers, Belgium), supplemented with cytokines and/or dexamethasone as indicated, for 6 days.

Following stimulators were used: human interferon (IFN)γ, from TEBU Peprotech (Frankfurt am Main, Germany), at a concentration of 1000 U/ml; IL-4 from TEBU Peprotech, at a concentration of 10 ng/ml; dexamethasone (Sigma) at a concentration of 1 × 10^−7^ M; TGF-β1 from Tebu Peprotch at a concentration of 10 ng/ml.

### Isolation of RNA and synthesis of cDNA

For RNA isolation, the cells were lysed directly in the plastic Petri dishes and the RNA was isolated using RNeasy Mini kit (Qiagen, Hilden, Germany) according to the recommendations of the manufacturer. Total RNA (2 μg) was treated with 2U RNase free DNase (Ambion, Austin, TX, USA) and used for Reverse Transcription (RT) with Superscript III reverse transcriptase (Invitrogen, Karlsruhe, Germany) using oligo dT primers according to the recommendations of the manufacturer. Obtained cDNA was diluted 1:10 with DDH2O and 1 μl was used for PCR reaction.

### Real-time PCR analysis

Real-time RT-PCR analysis of YKL-39 was performed using Hs00187790_m1 TaqMan^®^ assay (Applied Biosystems, Darmstadt, Germany). For normalization a house keeping index (HKI) was generated basing on the analysis of the expression of GAPD, ACTB and HPRT1 (Vandesompele et al. 2002). For the analysis of the expression of ACTB and HPRT1 ready TaqMan probes Hs03023880_g1 and Hs99999909_m1 respectively were used (Applied Biosystems). The assay for GAPDH consisted of following oligonucleotides: sense primer 5′-CATCCATGACAACTTTGGTATCGT used at 900 nM, antisense primer 5′-CAGTCTTCTGGGTGGCAGTGA used at 300 nM and TaqMan^®^ probe JOE-AAGGACTCATGACCACAGTCCATGCC-BHQ1 used at 200 nM. Oligonucleotides were synthesized by MWG-Biotech (Ebersberg, Germany). The experiments were performed on ABI PRISM^®^ 7000 sequence detection system (Applied Biosystems) using standard conditions. Relative quantification of the expression levels of analysed gene was performed using ΔΔCt method. For standard curve generation 5 serial dilution points of a PCR product, containing target sequence ranging from 10^3^ to 10^8^ copies were used. The efficiencies of all reactions were above 95%.

### Statistics

Statistical analysis of real-time RT-PCR results was performed using ANOVA function of Excel program of Microsoft^®^ office XP.

## Results and Discussion

Several Glyco_18 domain containing proteins, which comprise family of mammalian chitinases and chitinase-like proteins, were found to be abundantly expressed by macrophages both in human and in rodents. However, chitinase-like proteins YKL-39 was only found to be expressed by synovial fibroblasts during osteoarthritis (OA) (Knorr et al. 2003; Steck et al. 2002). During arthritis, monocytes and macrophages are attracted to the sites of inflammation due to high levels of galectin 3 (Karouzakis et al. 2006). Further macrophages are exposed to high levels of TGF-β. We addressed the question, whether TGF-β is able to induced YKL-39 expression in monocyte-derived human macrophages. Monocytes were isolated from buffy coats by sequential gradient centrifugation and enriched using CD14 MACS sorting to achieve over 95% of purity. Immediately after isolation monocytes were stimulated by single cytokines or their combinations. Following stimulations were used: IL-4; TGF-β; IL-4+TGF-β; IL-4+dexameth-asone; IL - 4 + dexamethasone + TGF - β; dexamethasone +TGF-β; IFNγ; IFNγ+TGF-β. After six days of differentiation in culture, cells were harvested and RNA was used for the quantification of YKL-39 expression level. Non-stimulated monocyte-derived macrophages cultivated for 6 days were used as a control. Since there are no commercially available anti-YKL-39 antibodies we evaluated YKL-39 expression regulation on the mRNA level.

**Figure 1:**
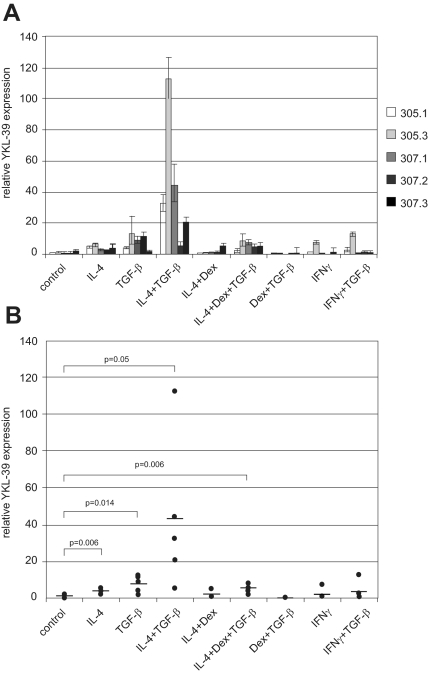
(**A**) Analysis of YKL-39 mRNA expression in primary human monocyte-derived macrophages. YKL-39 levels were normalized to GAPDH mRNA expression. Five individual donors are presented. Normalized expression level in control sample of donor 305.1 was taken as a 1. Stimulations were performed for 6 days in X-vivo medium. **B**) Statistical analysis of the effects of different stimuli on the expression of YKL-39 in primary human monocyte derived macrophages. Dots represent expression levels obtained for individual donors. Mean values are indicated as lines. p-values were obtained using ANOVA.

Real-time PCR analysis revealed, that TGF-β alone has stimulatory effect of 4.1 to 12.7 times in four out of five donors tested (Fig. 1A, Table 1). This effect was statistically significant with p = 0.014 (Fig. 1B). TGF-β is potent regulatory cytokine with diverse activities. Well established function of TGF-β in immune system is to maintain tolerance by controlling proliferation, differentiation, activation and survival of various immune cells (Li et al. 2006). TGF-β is produced by numerous cell types including T-and B-lymphocytes, NK cells, DCs, macrophages, mast cells, and granulocytes. In particular granulocytes may influence synovial effusion by production of TGF-β1. Potent source of high levels of TGF-β are tumour cells. During cancer progression tumour cells frequently lose the growth-inhibitory responses to TGF-β and instead benefit from high TGF-β levels (Bierie et al. 2006). In particular beneficial effects of high TGF-β levels can be due to modulation of phenotype of tumour associated macrophages (TAM) which help tumour to establish immunologically tolerogenic environment. Our data suggest that YKL-39 can be expected to be secreted by TAMs. Further examination for specific tumours is needed to consider YKL-39 as a potential biomarker.

In tumours, elevated TGF-β levels can correlate with high levels of IL-4. IL-4 is a potent immuno-modulatory cytokine secreted by T-helper 2 (Th2) lymphocytes, eosinophils, and mast cells. Beside tumours, high levels of IL-4 are present in tissues of patients with chronic inflammations, in particular in atherosclerosis (Lee et al. 2006). Next we investigated whether IL-4 has a stimulatory effect on YKL-39 expression. IL-4 alone showed weak stimulatory effect on the YKL-39 mRNA level in macrophages from all five donors investigated (lowest 1.9 times and highest 4.7 times) (Fig. 1A, Table 1). However the dramatic effect was observed if IL-4 was used in combination with TGF-β. Monocytes derived from four out of five donors responded to IL-4/TGF-β combination by 9.2 to 83.6 fold over-expression of YKL-39 mRNA (Fig. 1A, Table 1). Both, the effect of IL-4 alone and IL-4 in combination with TGF-β were statistically significant (p-values 0.006 and 0.05 respectively) (Fig. 1B). Thus IL-4 and TGF-β exhibit co-operative effect and modulate macrophages phenotype.

Our data suggest that monocytes recruited to the sites of chronic inflammation, are typically characterized by concomitant presence of IL-4 and TGF-β, can be a source of YKL-39. This cooperative action can be expected in case of many tumours, atherosclerosis and other types of chronic inflammation. Since chronic inflammatory conditions are frequently characterized by mixed immunological profile of Th1 and Th2 reactions, we decided to analyze the effect of IFNγ, prototype Th1 cytokine on YKL-39 expression. IFNγ alone did not have modulatory effect on YKL-39 expression, however it slightly inhibited the stimulatory effect of TGF-β (Fig. 1A, Table 1). It was recently demonstrated, that Th1 cells predominate in the synovium of patients with OA (Ishii et al. 2002). IFNγ seems to be prevailing cytokine in OA tissues, and IFNγ has inhibitory effect on the production of YKL-39 by monocyte-derived macrophages. Thus synovial fibroblasts likely represent the major source of YKL-39 in OA.

Glucocorticoids are universally used anti-inflammatory therapeutics. Next, we analyzed whether synthetic glucocorticoid dexamethasone, applied at therapeutic concentrations, has an effect on the induction of YKL-39 by IL-4 and TGF-β. We found, that dexamethasone significantly decreased stimulatory effect both for IL-4 and TGF-β stimulations as well as for the combined stimulation for most of donors (Fig. 1A).

In summary, our data indicate, that YKL-39 can be considered in future as a biomarker for specific macrophage activation with IL-4 and TGF-β, which can occur in tumours and chronic inflammations, and particularly in atherosclerosis. Generation of specific anti-YKL-39 antibodies is an urgent next step to evaluate the amount of YKL-39 protein produced by macrophages as well as quantification of YKL-39 protein in tissues and in circulation.

Further perspective is the evaluation of YKL-39 both on mRNA and protein level in neurode-generative diseases. Many evidences indicate that abnormal TGF-β signalling in neuronal cells contributes to the progression of Alzheimer diseases (AD). At the same time very recent data indicate that activation of microglia can have an essential role during Alzheimer disease progression. Microglia is a primary component of innate immune response in the brain and is associated with neuritic plaques in Alzheimer disease (AD). Type of microglial activation is currently a controversial issue. Recent data indicate that microglia in AD may exhibit a hybrid activation state that includes characteristics of classical and alternative activation (Colton et al. 2006). However the role co-operative effect of cytokines and TGF-β on microglia activation was not addressed. In the future YKL-39 can serve as a biomarker which indicates the direction of microglial polarisation.

## Figures and Tables

**Table 1:** Fold change of YKL-39 mRNA level in human peripheral blood-derived monocytes differentiated in culture. Stimulations are performed continuously for 6 days in serum-free X-vivo medium. Macrophages differentiated in culture for 6 days without additional stimulations are used as a control (1.0).

Stimulations	Individual donors				
	305.1	305.3	307.1	307.2	307.3
Control	1.0	1.0	1.0	1.0	1.0
IL-4	5.0	4.4	5.6	2.8	2.6
TGF-β	2.7	7.7	16.3	12.8	1.0
IL-4/TGF-β	33.8	86.9	75.9	7.1	11.8
IL-4/Dex	0.6	1.2	2.5	2.2	3.5
IL-4/Dex/TGF-β	2.5	6.3	16.8	6.4	3.7
Dex/TGF-β	0.5	0.1	0.0	0.4	0.2
IFNγ	0.8	2.5	0.6	0.1	0.4
IFNγ/TGF-β	1.8	5.0	0.7	0.9	0.3
